# The use of sequential window acquisition of all theoretical fragment ion spectra (SWATH), a data‐independent acquisition high‐resolution mass spectrometry  approach, in forensic toxicological regimes: A review

**DOI:** 10.1002/dta.3700

**Published:** 2024-05-09

**Authors:** Maria Sarkisian, Luke N. Rodda

**Affiliations:** ^1^ Forensic Laboratory Division Office of the Chief Medical Examiner San Francisco California USA; ^2^ School of Forensic Sciences Oklahoma State University, Center for Health Sciences Tulsa Oklahoma USA; ^3^ Department of Laboratory Medicine University of California, San Francisco San Francisco California USA

**Keywords:** best practices, comprehensive forensic toxicology, liquid chromatography–tandem mass spectrometry, QTOF/MS, sequential windowed acquisition of all theoretical fragment ion mass spectra (SWATH)

## Abstract

Sequential window acquisition of all theoretical fragment ion spectra (SWATH) is a type of high‐resolution mass spectrometry that uses data‐independent acquisition. Compared with more targeted acquisition schemes, the power behind this data‐independent acquisition technique comes from its ability to mitigate interferences via the use of SWATH acquisition windows (Q1 quadrupole isolation windows) while still obtaining all accurate mass information. However, consistent with high‐resolution mass spectrometry techniques, its routine and high throughput implementation in forensic toxicology is limited due to the complex processing power required to effectively manage the large amount of acquired data. It is therefore pivotal to create an efficient and validated identification criterion that confidently reports suspected positive detections as a confirmational technique for final reporting. This review examines all publications that implemented SWATH in a forensic toxicological framework with suggestive best practices and commonly used criteria. Seventeen publications were reviewed for extraction, liquid chromatography and mass spectrometry parameters, and more specifically for all SWATH applicable characteristics including spray voltages, collision energies and spreads, mass error, isotopic ratio difference, retention time error, and library score thresholds. Notwithstanding the challenges SWATH implementation faces for a laboratory, the technique demonstrates its potential to be utilized in routine forensic toxicology testing regimes and aids in the detection of both common and emerging novel drugs simultaneously.

## Graphical abstract

A review of 17 publications assessing SWATH in forensic toxicology was conducted, highlighting key parameters such as LC and MS settings, mass error, and library thresholds. Despite challenges, SWATH shows promise for routine forensic toxicology testing, enabling the simultaneous detection of conventional and emerging novel drugs.

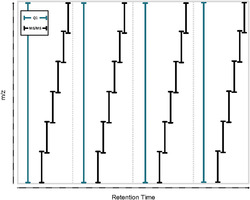

## 1 INTRODUCTION

Liquid chromatography coupled with high‐resolution mass spectrometry (LC–HRMS) can provide sensitive mass spectral data for the identification of known and unknown compounds. However, developing and implementing methods that use LC–HRMS techniques in routine analysis is found to be challenging due to perceived complexity. Immunoassays have historically been the primary method for drug screening due to low cost and the ability to provide rapid results; however, this methodology is accompanied by various limitations. Namely, in specificity, the identification of drugs mainly relies on drug class, as the assays are unable to determine the specific drug of interest.^1^, ^2^ Gas chromatography–mass spectrometry (GC–MS) methods prove to be a better screening method than immunoassays for certain drug classes, however require long sample preparation including derivatization which is a staff‐resource heavy technique. Liquid chromatography–mass spectrometry (LC–MS) methods also demonstrate a viable screening technique.^3^ Although efficient and sensitive, LC–MS faces limitations that stem mainly from the necessity of a targeted analysis, which will result in potential false negatives in casework. Moreover, the methodology requires optimization for targeting each specific analyte through the acquisition of individual reference materials.^4^ Apart from gas chromatography–mass spectrometry which possesses a better capability to perform general unknown screening of drugs and xenobiotics, these techniques do not allow for the retrospective interrogation of data to reveal previously undetected analytes.

Many of these limitations can be mitigated by implementing liquid chromatography quadrupole time‐of‐flight mass spectrometry. This LC–HRMS technique can adjust to the rapidly evolving landscape of illicit substances while also offering fast acquisition, high sensitivity, and resolution via high mass accuracy.^5^ Quadrupole time‐of‐flight (QTOF) is one of the most powerful tools in compound identification, due to its ability to measure the accurate mass of precursor and product ions, obtain isotope patterns, and determine retention times.^6^ Subsequently, forensic toxicologists can rapidly acquire chemical profiles, accurate mass information, and analyte‐specific tandem mass (MS/MS) spectra, enhancing their confidence in the identification of compounds.^7^ One of the main advantages to this high‐resolution acquisition is the capability to identify unknown compounds through accurate mass formula elucidation. During the acquisition process, the accurate mass of all precursors is measured, enabling the computation of the molecular formula. Additional fragmentation provides structural information, allowing for real‐time or retrospective library database searching.^4^

Two HRMS acquisition techniques can be used in order to obtain fragment (MS/MS) information: data (or information) “dependent” acquisition (DDA) and data “independent” acquisition (DIA).^8^ With DDA, MS/MS spectra are produced with minimal interference due to the narrow selection of precursor m/z windows. The isolating Q1 quadrupole transfers only the minimal range of precursor ions within the window to the collision cell, generating product ions exclusively derived from the selected precursor. This MS/MS acquisition is characterized by several predefined conditions, including whether the precursor ion is among a predefined number of most abundant ions or is included in a preset precursor ion list. While DDA can offer high‐quality MS/MS spectra, it may lead to false negatives and cannot be regarded as a true unknown screening technique. This limitation arises from its inability to capture fragmentation information for all precursor ions, irrespective of whether the precursor abundance criteria are met.^5^ Acquisition using DIA techniques is independent from any such preset conditions. There are two modes that are used with DIA, where either the single fragmentation of all precursor ions occurs or the m/z range is divided into smaller fixed windows that are each independently fragmented. All product ions are recorded regardless of their precursor ion properties. However, the resultant nonselective MS/MS spectrum may lack specificity, particularly in cases of coeluting compounds. This limitation can be partially addressed by applying chromatographic identification criteria to enhance selectivity.^5^

Sequential windowed acquisition of all theoretical fragment ion mass spectra (SWATH) represents a DIA acquisition mode in which fragmentation is performed on all precursor ions that enter the mass spectrometer. This is typically accomplished through isolated windows spanning 20–50 m/z across the entire mass range of interest. Ions within each window are transferred into the collision cell, where product ions are generated through the rapid cycling of low, medium, and high collision energies.^5^ Numerous repeated analyses of each SWATH window across a chromatographic peak yield a comprehensive precursor and fragment ion map, effectively digitizing each sample.^9^ In employing this nontargeted approach, there is no requirement for modifications to the acquisition method to accommodate the inclusion of new drugs within the precursor mass range, for example, when detecting emerging novel psychoactive substances (NPS). The data obtained can be retrospectively analyzed for unknown compounds,^1^ and the specificity of this acquisition type allows for preliminary structural elucidation of NPS even in the absence of a standard reference material.^10^

The focus for this review was specifically the implementation of the SWATH acquisition strategy within the forensic toxicology and similar communities. Due to the novelty of this technique, a holistic understanding for researchers and practitioners wanting to bring a technique like this into their laboratories is presented. It also lays the foundation for future advancements by highlighting gaps in knowledge and promoting collaborative efforts in refining and expanding the applications of SWATH‐MS in forensic toxicology.

## 2 REVIEW OF SWATH ANALYTICAL METHODS

A literature review was conducted by journal article searches through the Oklahoma State University Library online database and PubMed. Keywords used for the search were Quadrupole Time of Flight, Toxicology, QTOF, Forensic Toxicology, Sequential Window Acquisition, Data‐Independent Acquisition, SWATH. and Mass Spectrometry. Articles were only selected if they specified the use of SWATH and were utilized in a forensic toxicology or analogous setting. A total of 17 articles within the review's criteria and their described SWATH method were evaluated. The scope, sensitivity, sample application, extraction, and liquid chromatography parameters of the methods are described in Table 1. The mass spectrometry acquisition, processing, identification, and validation parameters are summarized in Table 2.

**TABLE 1:** Scope, sensitivity, sample application, extraction, and liquid chromatography parameters of sequential window acquisition of all theoretical fragment ion spectra methods.

Ref.	Scope	Target analytes	LOD (ng/mL)	Validated	Purpose	No. of analytes (validated)	Sample type	Extraction	Column	Injection vol. (μL)	Flow rate (mL/min)	Run time (min)
^1^	FTX	SCA	0.25–20	Yes	Qual	47 (47)	UR	SLE	Ultra Biphenyl HPLC (100 × 2.1 mm; 3 μm)	50	0.5	15
^4^	FTX	HAL, OPI, STI, NPS	0.5–50,000	Yes	Qual	137 (259)	BL, UR, PL, TS	LLE	Kinetex C18 (50 × 3.0 mm; 2.6 μm)	10	0.4	15.5
^2^	MET	ACE, OTH, BB	NP	No	NP	NP	UR	OTH	ReproSil‐Pur C18 (150 × 2.0 mm; 2.4 μm)	24	0.3	20
^7^	FTX	AMP, ANL, ANE, AED, AD, AH, AP, BZD, BB, CCB, CAR, COC, COX, DIS, MR, OPI, OTH, SCA, SNRI, SNDRI, STI, SCA, ZD	0.5–2500	Yes^a^	Qual	664 (151)	BL	PP	Kinetex Phenyl‐Hexyl (50 × 2.1 mm; 2.6 μm)	5	0.5	8.5
^8^	FTX	AD	NP	Yes	Qual/Quant	39 (33)	BL	PP	Synergi Polar RP (100 × 2.0 mm; 2.5 μm, 100A)	10	0.5	20
^9^	FTX	AMP, AD, AP, OTH, BZD, COC, STI, ZD	N/A	No	Qual	8	BL, UR, PL,	SPE	Gemini C18 (100 × 1 mm; 3 μm)	5	0.035	10
^10^	FTX	SCN	0.05–1	Yes	Qual	38 (38)	BL, UR	LLE; SPE	Kinetex C18 (50 × 3.0 mm; 2.6 μm)	20	0.5	7
^11^	CLN	HAL, OPI, SCA, SCN	0.04–2.4 (ng/g)	Yes	Qual/Quant	137 (137)	MEC	SPE	Kinetex C18 (100 × 2.1 mm; 1.7 μm)	5	0.5	20
^12^	FTX	AMP, SCA	1	Yes	Quant	400 (10)	OF	LLE	Kinetex C18 (50 × 3.0 mm; 2.6 μm)	10	0.4	15.5
^13^	CLN	SCA	50	No	Qual	1	UR	LLE	Zorbax Eclipse Plus C18 (100 × 2.1 mm; 1.8 μm)	5	0.3	14
^14^	CLN, FTX	AMP, ANL, ANE, AD, AP, BZD, COC, DIS, OPI, PDE, SCA, SCN, ZD	N/A	No	Qual	N/A	BL	LLE	Synergi Polar RP C18 (100 × 2.0 mm ID; 2.5 μm, 100A)	10	0.5	20
^15^	FTX	AMP, ANL, ANE, AED, AD, AH, BZD, CAN, MR, OPI, ZD	N/A	No	Qual	450	BL, UR	SPE; DS; PP	HPLC RP (50 × 2.1 mm)	10	1.5	6.5
^16^	FTX	AMP, AD, AH, AP, BZD, COC, OPI, ZD	0.5–600	Yes	Qual/Quant	24 (24)	BL	PP	Synergi Polar RP (100 × 2.0 mm; 2.5 μm, 100A)	5	0.5	12.2
^17^	CLN	AMP, ANL, AD, AP, BZD, CAN, CAR, SCA, COC, DIS, HAL, MR, OPI, ZD	5–100	No	Qual	81 (81)	UR	DS	Kinetex C18 (50 × 3.0 mm; 2.6 μm)	10	0.4	10
^18^	FTX	AED, AD, BZD, COC, DIS, OPI	NP	NP	NP	NP	DUS	OTH	Kinetex C8 (50 × 2.1 mm; 2.6 μm)	1	0.35	15
^19^	FTX	NPS, NSO, SCA, SCN, OPI	2–7.5	Yes	Qual	132 (132)	DBS	OTH	Kinetex C18 column (100 × 2.1 mm, 1.7 μm)	5	0.5	10
^20^	FTX	NSO	0.2–2.4 (pg/mg)	Yes	Qual	12 (12)	Hair	OTH	Kinetex C18 column (100 × 2.1 mm, 1.7 μm)	3	0.5	7

Abbreviations: ACE, ACE inhibitors; AD, antidepressants; AED, antiepileptics; AH, antihistamines; AMP, amphetamines; ANE, anesthetics; ANL, analgesics; AP, antipsychotics; BB, beta blockers; BL, blood; BZD, benzodiazepines; CAN, cannabinoids; CAR, cardiac; CCB, calcium channel blockers; CLN, clinical toxicology; COC, cocaine and metabolites; COX, COX inhibitors, DBS, dried blood spots; DIS, dissociatives; DS, dilute and shoot; DUS, dried urine spots; FTX, forensic toxicology; HAL, hallucinogens; LLE, liquid–liquid extraction; MEC; meconium; MET, metabolomics; MR, muscle relaxants; NP, not provided; NPS, novel psychoactive substances; NSO, novel synthetic opioids; OF, oral fluid; OPI, opioids; OTH, other(s); PDE, phosphodiesterase inhibitors; PL, plasma; PP, protein precipitation; Qual, qualitative; Quant, quantitative; SCA, synthetic cathinones; SCN, synthetic cannabinoid; SLE, supported‐liquid extraction; SNDRI, serotonin‐norepinephrine‐dopamine reuptake inhibitors; SNRI, serotonin‐norepinephrine reuptake inhibitors; SPE, solid‐phase extraction; STI, stimulants; TS, tissue; UR, urine; ZD, z drugs.

^a^ This information was provided through direct communication with the author.

**TABLE 2:** Mass spectrometry acquisition, processing, and identification parameters of sequential window acquisition of all theoretical fragment ion spectra (SWATH) methods.

Ref.^a^	MS^b^	Spray voltage (kV)	MS acc. time (MS)	MS/MS acc. time (MS)	Total cycle time (s)	SWATH windows	MS/MS m/z range	MS/MS window width(s)	MS/MS variable or fixed	Mass error (ppm)	IR diff. (%)	RT error^c^	Library score	Library search algorithm
^1^	T	4	100	25	0.9	30	228–408	6	F	≤15	≤80	≤2%	≥60	NP
^2^	T	5.2	50	25	1	37	40–1000	15–210	V	≤5	≤10	NP	≥70	CAND
^4^	T	2.5	NP	NP	0.77	28	100–510	6–34	V	≤10	≤50	<0.35 min	≥50	NP
^7^	Q	2.5	NP	NP	NP	14	100–650	22–121^d^	V	≤5	≤20	≤5%	≥60	NP
^8^	T	5.5	100	40	1.2	28	100–650	20	F	≤5	≤40	≤5%	≥60	CONF
^9^	T	4.5	50	36	NP	25	100–600	21	F	NP	NP	NP	NP	CAND
^10^	T	4	NP	NP	0.91	27	50–550	25	F	≤5	≤30	<0.25 min	≥70	NP
^11^	Q	3	NP	NP	0.564	12	150–465	25^d^	V	≤5^d^	≤2.5^d^	≤2.5%^d^	≥70^d^	NP
^14^	T	5.5	100	40	1.2	28	100–650	20	F	≤5	≤40	≤5%^d^	≥60	CONF
^15^	T, Ti	2.5	100	25	0.575/ 0.525	17/15^e^	40–1000	40/V^e^	F/V	≤5	Not used	≤3%	≥70	NP
^16^	Tii	5.5	50	25	0.6	22^d^	100‐650	25	F	≤20^d^	≤15^d^	≤5.4%^d^	≥70^d^	NP
^17^	T	5.5	NP	NP	1	30	100–650	18.5/6–59	F/V	NP	NP	NP	≥70	F/P
^18^	T	NP	NP	NP	0.86	25	50–950	35	F	≤5	≤10	≤5%	≥70	NP
^19^	Q	NP	100	40	0.933	18	50–575	NP	V	NP	NP	NP	NP	NP
^20^	Q	NP	NP	NP	0.555	12	230–450	NP	V	≤5	NP	NP	NP	NP

Abbreviations: acc., accumulation; CAND, candidate; CONF, confirmation; diff., difference; F, fixed MS/MS window; F/{, fit or purity; IR, isotopic ratio; NP, not provided; Q, QTOF X500R; RT, retention time; T, TripleTOF 5600+; Ti, TripleTOF 4600; Tii, TripleTOF 6600; V, variable MS/MS window.

^a^ Krotulski et al.^12^ and Mazzarino et al.^13^ did not include detailed identification parameter and SWATH window information, therefore, were excluded from this table.

^**b**^ Collision energy and collision energy spread were 30 and 15, respectively, for all methods except Klont et al., ^2^ who had a collision energy of 40 and a collision energy spread of 30.

^c^ All references used a retention time error percent except for Whitman et al.,^17^ which used a retention time error delta.

^d^ This information was provided through direct communication with the author(s).

^e^ Seventeen fixed sized SWATH windows (40 m/z each) for blood and 15 variable sized SWATH windows utilized for urine.

### 2.1 Drug scope

The target compounds throughout each study varied in terms of quantity, drug class, and limits of detection. Within scope, drug classes were varied and included amphetamines, analgesics, anesthetics, antiarrhythmics, anticonvulsants, antidepressants, anthelmintics, antihistamines, antipsychotics, barbiturates, benzodiazepines, cannabinoids, cathinones, COX inhibitors, esters, muscle relaxants, opiates, sedatives, stimulants, synthetic cathinones, synthetic cannabinoids, and nonbenzodiazepines (Z‐drugs). Three out of the 17 studies solely focused on NPS,^4^, ^11^, ^12^ one solely on antidepressants,^8^ and two on synthetic cannabinoids.^1^, ^10^ The most prevalent of all drug classes were amphetamines, antidepressants, benzodiazepines, NPS, stimulants, and opioids as they were included in at least half of all studies analyzed. The quantity of target analytes varied between studies, with a minimum of one^13^ and a maximum of 664,^7^ and with limits of detection ranging from 0.05 to 50,000 ng/mL.

### 2.2 Sample type and extraction

Matrices analyzed were blood,^4^, ^7^, ^8^, ^9^, ^10^, ^14^, ^15^, ^16^ urine,^1^, ^2^, ^4^, ^9^, ^10^, ^15^, ^17^ plasma,^4^, ^9^ tissue,^4^ meconium,^11^ dried urine spots,^18^ oral fluid,^12^ dried blood spots,^19^ and hair.^20^ Blood was the most frequent sample type, included in 8 out of 17 studies (47%). Extraction types were liquid–liquid,^4^, ^10^, ^12^, ^14^ solid‐phase,^9^, ^10^, ^11^, ^15^ dilute and shoot,^15^, ^17^ protein precipitation,^7^, ^8^, ^15^, ^16^ supported liquid extraction,^1^ or other methods that were specific to dried spots^18^, ^19^ or hair.^20^ The most common extraction types were solid‐phase, protein precipitation, and liquid–liquid extractions included in four, four, and five studies, respectively.

### 2.3 Liquid chromatography and mass spectrometry parameters

Liquid chromatography systems used were the Shimadzu Prominence LC‐20ADXR HPLC,^1^, ^17^ UltiMate 3000 Rapid Separation Liquid Chromatography System,^8^, ^14^, ^16^ Eksigent 425,^9^ Dionex Ultimate 3000,^2^, ^18^ Shimadzu Nexera,^4^, ^10^, ^12^, ^15^ and SCIEX ExionLC AC.^7^, ^11^, ^19^, ^20^ Column types were C18, biphenyl, and phenyl‐hexyl, with the most common being C18. Typical reagents used for mobile phase A were ammonium acetate, acetic acid, acetonitrile, formic acid, ammonium formate, and water. Typical reagents used for mobile phase B were acetonitrile, acetic acid, formic acid, and methanol. Injection volumes ranged from 5 to 20 μL, column temperatures from 25°C to 45°C, flow rates from 0.02 to 1.5 mL/min, and run times from 6.5 to 40 min. There are a limited number of mass spectrometry instruments available that utilize SWATH. Sciex's TripleTOF 5600+ system was used in 11 of the studies (64%). The TripleTOF 4600, TripleTOF 6600, and X500R systems were also used, albeit with fewer published studies.

### 2.4 Acquisition and data analysis

Spray voltages (kV) ranged from 2.5 to 5.5, with 5.5 the most prevalent. All studies except one^2^ used a collision energy (eV) of 35 and a collision energy spread (eV) of 15. The number of SWATH windows ranged from 5 to 37, with m/z ranges starting as low as 30 m/z and ending as high as 1000 m/z with median (and most common) lower and higher brackets of 100 (100, *n* = 7) and 650 (650, *n* = 4), respectively. SWATH windows can either be fixed or variable, the use of which was evenly distributed in this review. Mass error, isotopic ratio difference, retention time error, and library scores were used in the identification criteria of all studies. The most common acceptance criteria for mass error (ppm) were ≤5. Isotopic ratio difference (%) acceptability varied between studies, ranging from ≤2.5 to ≤80. Acceptable retention time error ranged from ≤2% to ≤30% and <0.25 to <0.35 min, with the majority of the studies using a percent error between ≤2 and ≤5. One study used the absolute retention time error for their acceptance criteria.^17^ For library scores, a score of ≥70 was most common (41%). Data processing software used were AnalystTF, MultiQuant, Sciex OS, PeakView, and MasterView software, with the last two utilized in conjunction in 12 of the studies (70%).

### 2.5 Library optimization, search parameters, and other criteria

Due to the complexity of mass spectral data produced by SWATH, some papers used additional data and library criteria for identification. Roemmelt et al. used a confirmation‐based library search with an intensity threshold of 1%.^8^ Coupled with their defined library score and identification criteria, Scheidweiler et al. also required at least two common fragments in the sample and reference MS/MS for identification.^1^ Whitman et al. used a purity‐based library search and required a precursor ion intensity >1000 cps and two or more fragment ions for compound identification. They also conducted an experiment to compare and optimize both purity and fit library searching and found that the fit library search was the most sensitive for identifying compounds via SWATH.^17^ Krotulski et al. included a minimum intensity of >800 cps, a signal‐to‐noise ratio >10, acceptable chromatography, acceptable peak shape, and an acceptable comparison between library spectrums and controls (blanks) in their identification criteria.^4^, ^10^ The characteristics that deemed the chromatography and peak shape acceptable, however, were not described. The selection of the reference library, whether it is internally developed, supplied by a vendor, or obtained from online resources, can influence identification. Scheidweiler et al. built extracted‐ion chromatograms (EIC/XIC) for their target analytes using 10‐mDa‐wide extraction widths for all ions.^1^ Negri et al. built a library with information‐dependent acquisition spectra for each compound of interest.^7^

### 2.6 Validation

Method validation was conducted in 10 of the 17 studies (58%). All validations performed qualitative assessments, namely, specificity, sensitivity, recovery, matrix effects, and efficiency. Four methods also underwent quantitative validation. Guidelines used for validation were as follows: “Requirements for the validation of analytical methods” by Peters et al.,^21^ ANSI/ASB 036 “Standard practices for method validation in forensic toxicology,”^22^ “Experimental and statistical protocol for the effective validation of chromatographic analytical methods” and “Effective validation of chromatographic analytical methods: The illustrative case of androgenic steroids” by Alladio et al.,^23^, ^24^ ISO/IEC 17025, and the World Anti‐Doping Agency guidelines. While the ANSI/ASB standards are well recognized, the Alladio et al. protocol was used specifically for robust statistical analysis. ISO/IEC 17025 and the World Anti‐Doping Agency guidelines were used in conjunction for the validation by Mazzarino et al.^13^

### 2.7 System maintenance and performance

An automated calibration device system can be used in run to maintain MS calibration. Studies stated they ran a calibration device system calibration every five samples,^16^ every 10 samples,^5^ every four samples in a batch,^8^ and every 2 h within a run which is equivalent to approximately eight samples.^1^ Roemmelt et al. monitored the daily performance of the instrument with a test solution injected of five triazine compounds, where peak areas and retention times were plotted and compared with previous days injections to track and easily identify instrument fluctuations and possible performance issues.^8^ To monitor instrument performance in another study, positive and negative controls were analyzed after each batch of 20 extracts.^4^ Klont et al. used stable isotope‐labeled standards within each batch to monitor analytical performance as well as guide decision‐making for reanalyzation needs.^2^ These samples also aided in retention time shift assessments and monitoring of signal intensity differences between batches.

## 3 SWATH CHALLENGES

### 3.1 Interferences

Interferences pose a significant hurdle in data analysis as analytes that share product ions may belong to similar drug classes or fall within the same mass and retention time window, complicating the deconvolution of spectra during data analysis, particularly when coelution occurs. This is also true for SWATH windows containing numerous compounds. With compound identification partially weighed by library scores, background fragments due to coeluting compounds can lower confidence scores and cause false negatives.^17^ However, this problem can be mitigated by creating customized variable‐sized mass windows. This strategy was used by Zhang et al., who created an in‐house software that generated variable windows based on balancing the distribution of either the precursor ion population or total ion current.^25^ By doing this, SWATH and quadrupole selectivity were increased, resulting in cleaner MS/MS spectra and enabling lower limits of detection.^1^ Similarly, Whitman et al. optimized their SWATH windows to separate compounds with the same fragment ions into different windows.^17^ Regarding target interferences, Krotulski et al. discovered an interference between lidocaine and norfentanyl, where the lidocaine interfered with the M + 2 isotope of norfentanyl, resulting in a failed identification. This occurred inconsistently due to natural chromatographic behavior and only when lidocaine concentrations were significantly higher than that of norfentanyl. The coelution of compounds can also result in mass‐measurement errors, ultimately affecting the identification criteria and can result in false negatives.

**FIGURE 1:**
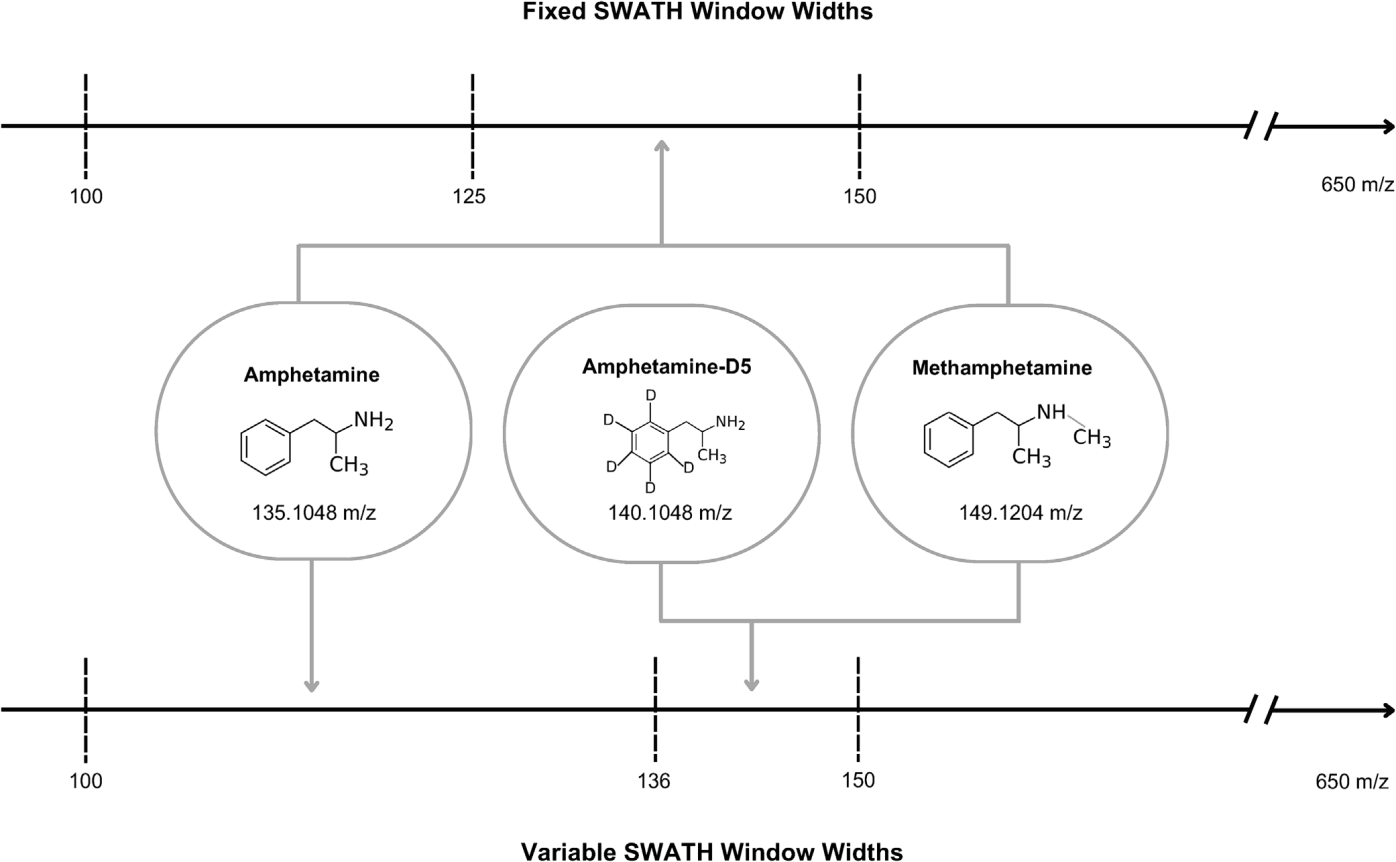
Implementation of fixed sequential window acquisition of all theoretical fragment ion spectra (SWATH) window widths, resulting in amphetamine, deuterated amphetamine, and methamphetamine falling within the same window—increasing the risk of tandem mass spectrometry interferences. Conversely, tandem mass spectrometry data of compounds are obtained in different windows using variable SWATH window widths—facilitating their distinct isolation and minimizing potential interferences.

The selection of internal standards has the potential to introduce interference. If deuterated compounds fall into the same SWATH window as their undeuterated analog, relative abundances in MS/MS spectra can be impacted.^8^ This problem can be mitigated via the optimization of SWATH windows by ensuring that deuterated compounds and undeuterated analogs do not fall in the same window. Figure 1 demonstrates the differences in using either set‐width or variable SWATH windows, showing the need for compounds and their deuterated counterparts to be in separate MS/MS windows and different set window widths enabling mitigation of MS/MS data interferences. Deuterated internal standards should be tested for MS/MS interferences during method development and validation. Further, related target compounds that share common fragments and coelute may also benefit from separate SWATH window analysis to mitigate MS/MS interferences.

### 3.2 Acquisition

Some compounds or drug classes may not ionize efficiently. Scheidweiler et al. noted that PB‐22 N‐hydroxypentyl‐3‐carboxyindole and PB‐22 N‐pentanoic‐3‐carboxyindole had poor ionization in positive mode, resulting in poor signal intensities and irreproducible spectra that erratically met the defined library criteria. To challenge this, they employed alternative MS/MS spectra criteria by monitoring four fragments specific for these analytes and requiring three fragments present for a positive identification.^1^

Although most of the referenced studies acquired data in positive ionization mode, negative ionization mode may also be required, particularly in methods with a focus on barbiturates or more acidic compounds. Incorporating both positive and negative ionization modes in SWATH can provide a comprehensive view of analytes; however, developing and optimizing a method that encompasses both modes poses a challenge due to the distinct requirements and potential interferences associated with each mode. Klont et al. developed acquisition methods in both positive and negative modes but used different LC columns and aqueous mobile phases for each ionization mode.^2^ The specific requirements that may be associated with each mode can further complicate the instrumentation and workflow, presenting a challenge for laboratories with high throughput and routine analysis needs.

### 3.3 Data interpretation

Efficient data interpretation in the context of SWATH acquisition poses a considerable challenge due to its untargeted approach and subsequently results in the generation of exceptionally large data files.^26^ For laboratories with limitations in computer power and storage, this becomes a significant impediment. A potential resolution to this challenge is proposed by Krotulski et al., who advocate for a targeted data processing strategy.^4^ In this approach, analyte identification criteria are fine‐tuned to optimize data analysis, resulting in a more comprehensive understanding within a shorter processing time. The targeted data processing method relies on referencing a predefined list of masses and a library database for positive analyte identification, incorporating criteria such as mass error, retention time error, isotope difference, and library score.^4^ On the contrary, untargeted processing can prove to be labor‐intensive and time‐consuming,^4^ posing a particular challenge for routine forensic testing laboratories. To make untargeted processing more viable for routine implementation, further developments are necessary to address current limitations and enhance its feasibility in everyday forensic settings.

## 4 SWATH ADVANTAGES

### 4.1 General unknown screening

SWATH proves to be a highly advantageous general screening technique, particularly when contrasted with DDA methods. The potential inconsistency of DDA methods, even after optimization, can lead to the incorrect omission of relevant ions.^8^ Unlike DDA methods, which limit the acquisition of all possible precursor ions by imposing a minimum threshold for ion selection, SWATH's precursor ion selection is nonrestrictive. Consequently, SWATH has the potential to identify more compounds at lower concentrations, making it generally more sensitive than DDA methods.^9^ The utilization of Q1 isolation windows in SWATH facilitates more selective MS/MS data acquisition, thereby significantly enhancing data quality compared with other DIA fragmentation techniques.^26^ To assess differences in matrix effect tolerance and sensitivity between DDA and SWATH, Arnhard et al. analyzed authentic samples using both acquisitions. This resulted in the positive identification of 218 compounds with DDA and 272 compounds with SWATH, underscoring that DDA tends to yield less correct positive identifications than SWATH and is more susceptible to effects from interferences.^9^ Additionally, SWATH acquisition enables nontargeted data processing, wherein the software extracts peaks not included in the targeted screening of the processing method. The software not only provides exact precursor mass information but also offers suggestions for potential formulas.^8^

### 4.2 Testing of unknown and new drugs including NPS

A great analytical challenge in the field of forensic toxicology is the analysis of NPS. Given the diverse chemical classes within NPS and their rapid emergence in the community, targeted analytical methods must continually adapt to identify these compounds during drug screening. The application of a DIA technique, such as SWATH, addresses this challenge by allowing untargeted data acquisition. This approach creates a more comprehensive digital map of the sample, facilitating retrospective data analysis for newly emerging compounds. These compounds can be identified through data reinterrogation, typically when reference standards become available.^1^

SWATH high‐resolution methods offer increased flexibility in incorporating new compounds as they emerge. Unlike LC–MS/MS applications where infusion optimization or the addition of analyte transitions to the acquisition method is necessary, SWATH does not require these adjustments. For qualitative screening or confirmation methods, the inclusion of new compounds within the targeted processing method should undergo validation studies encompassing specificity, sensitivity, extraction efficiency, matrix effect, stability, and carryover.^1^

## 5 SWATH BEST PRACTICE RECOMMENDATIONS

### 5.1 Data acquisition

The selection of an appropriate extraction method is contingent upon both the matrix type (e.g., blood and urine) and the various drug classes targeted. Different matrices require tailored approaches to efficiently extract and prepare analytes for subsequent analysis. Moreover, the chemical properties and characteristics of distinct drug classes may necessitate diverse extraction techniques. In the context of SWATH acquisition, which is inherently untargeted, it is advisable to align the extraction method with the untargeted nature of the analysis. This entails employing extraction methods that are broad in scope and capture a wide range of compounds. The synergy between untargeted SWATH acquisition and a compatible untargeted extraction approach contributes to a more inclusive and thorough assessment of the sample, especially in the dynamic and evolving landscape of forensic toxicology. It must be noted, however, that a more untargeted extraction can lead to more prominent matrix effects. Stationary phases for liquid chromatography must be considered in conjunction with the target analyte groups and extraction techniques. The most common columns used in this review were C18 due to their versatility with general unknown screening. If the target compounds include those of various polarities, biphenyl columns may also be considered. Optimization of extraction methods and columns, along with careful consideration of matrix effects and interferences, is essential during method development and validation.

Mass spectrometer‐specific parameters such as collision energy, collision energy spread, and in‐source fragmentation must be considered. SWATH acquisition utilizes collision‐induced dissociation (CID), where collision energies can significantly influence the spectra created.^27^ At a lower collision energy, parent ions are the most abundant, with fragmentation increasing at higher collision energies. SWATH isolation windows have been considered during CID optimization.^28^ Once the appropriate CID is established during method development, it is important to maintain consistency in these parameters for library acquisition, validation, and implementation.

The determination of the SWATH window range relies on the specific analytes targeted. In fields that target heavier molecules, such as proteomics, opting for a broader m/z range is a logical choice. In forensic applications, typical targeted compounds fall within the range of 50–600 m/z. If targeting a specific drug class, the choice of the m/z range should be restricted to encompass only the relevant values for that specific class. Maintaining a delicate balance in SWATH window widths is crucial; they should not be too wide, as this can result in less selective MS/MS spectra, nor too small, which can result in an increase in cycle time and decreased points across the peak.^8^ As observed in this review, the most common acquisition window width ranged between 20 and 25 m/z.

Guidelines pertaining to HRMS should be considered during the validation of any forensic toxicological method, including those utilizing SWATH acquisition. Although there are no specific validation guidelines exclusively tailored to SWATH acquisition, it is prudent to adhere to the currently established principles for validating new analytical methods in the field. ANSI/ASB Standard 036 Standard Practices for Method Validation in Forensic Toxicology^22^ is recommended for this purpose and has been specifically referenced in five of the reviewed publications.^4^, ^10^, ^11^, ^12^, ^19^ Although the remaining studies did not reference this standard, 10 of the 17 studies outlined validation criteria encompassing precision, accuracy, sensitivity, specificity, carryover, and processed sample stability.

### 5.2 Data processing

The primary identification criteria in processing SWATH data results are mass error, isotopic ratio difference, retention time error, and library match. Each parameter provides individual scores generated through the processing method and is adjusted to attain a combined weight score (CWS) that ranges from 0 to 100. The CWS is dependent on the percentage weight given to each parameter and serves to assist in faster data processing, mitigate false negatives, and ensure reliable positive identification of compounds. He et al. recommend a CWS acceptability threshold of 70, where the library score is >30, mass error <5 ppm, and RT <3.^15^ The CWS should be tested for efficiency, sensitivity, and specificity, as demonstrated by Colby et al. who provide a valuable guide specific to identification criteria using QTOF.^29^ This publication reported a detailed process used to determine the appropriate positivity criteria for HRMS screening procedures with an empirical approach. Using their comprehensive drug screen of 169 compounds with a QTOF‐MS, they found that a combined score of a threshold of 70, with a library match weight of 70%, mass error of 10%, retention time error of 10%, and isotope pattern difference of 10%, provided a drug identification efficiency rate of 99.2%. The results demonstrated the importance of library matching and the value of product ion spectra when it comes to accurately identifying compounds. Colby et al.'s publication was referenced by 1 of the 17 studies in this review.^17^ The outcome of a library match may vary depending on the search algorithm employed. In SCIEX software, three algorithms are available: candidate search, confirmation search, and smart confirmation search. Candidate search relies solely on spectral library matching, confirmation search matches the component name against the library, and smart confirmation first attempts to match by component name, resorting to spectral matching if no initial match is found. Both candidate and confirmation searches were used in the reviewed papers. Due to their potential impact on generated library scores, the library search algorithm chosen should also undergo assessment. All criteria related to data interpretation and acceptability should be validated and determined based on the purposes of the method. A summary of current practices based on the reviewed articles is provided in Table 3.

**TABLE 3:** Common parameters for mass spectrometers utilizing sequential window acquisition of all theoretical fragment ion spectra (SWATH) acquisition established from published literature.

Mass spectrometer	Spray voltage (kV)	MS/MS m/z range	SWATH window width	Variable or fixed window?	Mass error (ppm)	Isotopic ratio difference (%)	Retention time error (%)	Library score	Papers reviewed (*N*)
TripleTOF 5600+	5.5	100–650	20–25	Both	≤5	≤10 to ≤40	≤2 to ≤5	≥60 to ≥70	10
X500R	2.5 or 3	100–650	20–25	Variable	≤5	≤2.5 to ≤5	≤2.5	≥70	4

*Note*: Three publications were not included in this table as one did not use either of the mass spectrometers listed and the others did not include detailed information on identification parameters and SWATH windows. ^12^, ^13^, ^16^

ANSI/ASB Standard 113, titled “Standard for Identification Criteria in Forensic Toxicology,” provides a point system for the minimum requirements in the identification of an analyte in forensic toxicology laboratories.^30^ A minimum of 4 points must be reached per specific matrix with no more than three different techniques (hyphenated techniques count as one). When using an HRMS method such as SWATH, this 4‐point criteria is met. ANSI/ASB Standard 098, titled “Standard for Mass Spectral Data Acceptance in Forensic Toxicology,” focuses on data acquired using low‐ or high‐resolution mass spectrometers utilizing various ionization processes.^31^ Acceptance requirements for multiple‐stage mass analysis with HRMS include monitoring a minimum of one diagnostic product ion that shall meet a predefined mass error and isotopic distribution tolerance defined during method validation. If more than one product ion is monitored, the ratios should match that of reference material within acceptable tolerance ranges, or the spectrum shall be assessed through a library search and pass acceptance criteria defined through method validation. ANSI/ASB Standards 113 and 098 were not referenced by the published studies assessed in this review; however, they are guidelines that should be utilized during the implementation and validation of HRMS techniques, such as SWATH. Although these guidelines were not referenced, many of the studies reviewed in this paper met the minimum criteria described.

## 6 CONCLUSION

With the wide range of variables to consider in SWATH acquisition and processing, the selection of library parameters and the establishment of acceptable identification criteria are tied to the intended use and purpose of the method. Notably, validated and stricter criteria become imperative when the technique serves as a confirmational approach and for the final reporting of results. The absence of specific SWATH recommendations or guidelines is evident, reflecting the novelty of this technique and the emergence of peer‐reviewed publications in the field. The review uncovered notable gaps in the information provided by journal articles that are integral to the development of such a novel technique. To advance the utility and reliability of SWATH in forensic toxicology, further research is recommended, and more so should the technique be used for the final reporting of substances (e.g., confirmation). Studies should examine the functionality of SWATH, focusing on enhancing confidence parameters, refining identification criteria, and automating data processing to ultimately establish a consensus on best practices. While SWATH acquisition maintains many challenges consistent with other HRMS techniques, its incorporation into routine forensic work is underscored as a valuable and worthwhile investment. The ongoing exploration and refinement of SWATH methodologies in the field will contribute to the evolution of robust and standardized practices, bolstering its effectiveness and applicability in routine forensic analyses.

## Data Availability

The data underlying this article are available in the article may be provided upon request of specific data to the corresponding author.
